# Comprehensive Statistical Exploration of Prognostic (Bio-)Markers for Responses to Immune Checkpoint Inhibitor in Patients with Non-Small Cell Lung Cancer

**DOI:** 10.3390/cancers14010075

**Published:** 2021-12-24

**Authors:** Stefanie Hiltbrunner, Meta-Lina Spohn, Ramona Wechsler, Dilara Akhoundova, Lorenz Bankel, Sabrina Kasser, Svenja Bihr, Christian Britschgi, Marloes H. Maathuis, Alessandra Curioni-Fontecedro

**Affiliations:** 1 Department of Medical Oncology and Hematology, University Hospital Zurich, Rämistrasse 100, 8091 Zurich, Switzerland; stefanie.hiltbrunner@usz.ch (S.H.); dilara.akhoundovasanoyan@dbmr.unibe.ch (D.A.); lorenz.bankel@usz.ch (L.B.); sabrina.kasser@uzh.ch (S.K.); svenjabihr@web.de (S.B.); christian.britschgi@usz.ch (C.B.); 2 Comprehensive Cancer Center Zurich, University Hospital Zurich, Rämistrasse 100, 8091 Zurich, Switzerland; 3 Department of Mathematics, Seminar for Statistics, ETH Zurich, Rämistrasse 101, 8092 Zurich, Switzerland; metalina.spohn@stat.math.ethz.ch (M.-L.S.); ramona.wechsler@hotmail.com (R.W.); marloes.maathuis@stat.math.ethz.ch (M.H.M.)

**Keywords:** immune checkpoint inhibitors, non-small cell lung cancer, biomarker, basophils, statistical analysis

## Abstract

**Simple Summary:**

Metastatic non-small cell lung cancer (NSCLC) patients treated with immune checkpoint inhibitors (ICIs) may suffer from heavy side effects, and not all patients benefit from the treatment. Therefore, it is crucial to gain knowledge about possible (bio-)markers for response to ICIs. We used retrospective data acquired from NSCLC patients treated with ICIs in first- or further-line therapy settings, including 16 possible markers. We conducted a comprehensive statistical analysis study to find markers for response to treatment, assessed the robustness of our results, and discussed often encountered statistical pitfalls. Our study yielded hypotheses for various predictive and prognostic (bio-)markers for response to ICIs in NSCLC patients. In particular, we found that high basophil counts may be predictive for treatment response in patients in further-line therapy settings.

**Abstract:**

Metastatic non-small cell lung cancer (NSCLC) patients treated with immune checkpoint inhibitors (ICIs) may suffer from heavy side effects and not all patients benefit from the treatment. We conducted a comprehensive statistical analysis to identify promising (bio-)markers for treatment response. We analyzed retrospective data from NSCLC patients treated with ICIs in first- or further-line therapy settings at the University Hospital Zurich. We investigated 16 possible prognostic markers with respect to overall survival, tumor size reduction, and the development of an immune-related adverse event (irAE) and assessed the robustness of our results. For the further-line patient group, the most significant result was that increased basophil counts were associated with increased odds of tumor size reduction within three months and with the development of an irAE. For the first-line patient group, the most significant results were that increased lymphocyte counts, the histology of adenocarcinoma, and the intake of non-steroidal anti-rheumatic drugs (NSAR) were associated with decreased hazards of dying. Our study yielded new hypotheses for predictive (bio-)markers for response to ICIs in NSCLC patients. The possibly beneficial role of high basophil counts is a particularly interesting finding. Our results should be tested on independent data in a prospective fashion.

## 1. Introduction

Non-small cell lung cancer (NSCLC) is one of the most frequently diagnosed cancers worldwide. Approximately 70% of the patients are diagnosed with advanced and unresectable tumors with limited treatment options [1]. Platinum-based chemotherapy was the standard treatment until 2014 with only modest response rates and limited survival. The approval of immune checkpoint inhibitors (ICIs) has changed the treatment strategies for advanced NSCLC and significantly improved response rates and survival of patients [2]. ICIs, such as pembrolizumab, nivolumab (both anti-PD-1 antibodies), and atezolizumab (anti-PD-L1 antibody), are approved for the treatment of NSCLC. Nevertheless, patients may suffer from heavy side effects, and clinical effectiveness of ICIs has been shown to be limited to a subgroup of patients [3]. Thus, improving the knowledge on prognostic and predictive (bio-)markers is crucial for future patient selection and to stratify patients in future clinical trials.

A number of blood and tumor markers have been evaluated in clinical studies, such as PD-L1 expression in the tumor cells (PD-L1TC), tumor mutational burden (TMB), neutrophil to lymphocyte ratio (NLR), the presence of tumor-infiltrating lymphocytes (TIL) (such as CD8^+^ T cells), or a specific immune gene signature correlating with response [4,5,6,7,8]. An expression of PD-L1TC of over 50% is widely used for decision to treatment with ICIs, but patients can still respond if PD-L1TC is below this threshold, or not respond even if PD-L1TC is high [9]. Many markers are still controversial, due to high inter- and intra-tumor heterogeneity [10] and high variability in expression levels due to different storage of samples [11]. Furthermore, the field lacks standardization of different medical platforms; in particular, PD-L1 immunohistochemistry assays differ in their sensitivity and specificity leading to different outcomes [12]. In addition, there is no broadly applied standardization of the statistical analyses for observed data, including a critical discussion of the results. The choice and interpretation of statistical tools heavily influence the read out of results and can induce bias in marker discovery, leading to incorrect and non-replicable conclusions [13]. All these factors make it difficult to find verifiable prognostic markers that can be adapted for use in the clinical setting.

In this work, we applied the REMARK guidelines for tumor marker prognostic studies [14]. The REMARK guidelines consist of a checklist of items, which should be reported in prognostic tumor marker studies. We conducted an exploratory statistical analysis study to detect promising (bio-)markers predictive for three outcomes of patients undergoing treatment with ICIs: overall survival, tumor size reduction within the first three months of treatment, and the development of an immune-related adverse event. Our study evaluated 16 clinical and tumor markers collected during clinical routine in patients with stage IV NSCLC, treated with anti-PD-1/anti-PD-L1 checkpoint inhibitors as the first- or further-line of therapy. We discuss the reliability and sensitivity of our results with respect to model assumptions, multiple testing correction, and influential observations.

## 2. Methods

### 2.1. The Two Patient Cohorts

We retrospectively collected clinical data from 182 stage-IV NSCLC patients (adenocarcinoma and squamous cell carcinoma) who received treatment with anti-PD-1/PD-L1 checkpoint inhibitors (nivolumab, pembrolizumab or atezolizumab) at the University Hospital Zurich (USZ) as standard of care between 2015 and 2019. Treatment with ICIs and chemotherapy was applied in the first- or further-line of treatment. These two patient groups (first- and further-line patient group) are inherently different (Table 1). Therefore, we analyzed these groups separately. Within both groups, patients were treated either with ICIs alone or in combination with chemotherapy (ICI + Chemo). All patients included in the analyses consented to the study. The study was approved by the Cantonal ethical committee of Zurich (KEK-ZH-2018-01919) and performed in accordance with the declaration of Helsinki.

We investigated possible (bio-)markers with respect to how predictive and prognostic they are for three different responses to ICIs, which can be divided into two categories: (i) binary responses, consisting of two outcomes (yes, no), including response to treatment within the first three months (Response3mt) and the development of immune-related adverse events (irAE); and (ii) overall survival calculated from the start of ICI therapy. Response3mt was determined by radiological assessment, according to the response evaluation criteria in solid tumors (RECIST), performed after three months of therapy using PET/CT imaging [15], where we treated complete response and partial response within the first three months of ICI therapy as “yes”, and stable disease and progressing disease within the first three months of ICI as “no”. For irAE, we reported whether an immune-related adverse event occurred during ICI therapy (yes, no). Finally, the overall survival time can be right-censored. Its information is recorded as the minimum of the duration of follow-up and the duration of survival (OS.cen), combined with the indicator of death (yes, no), which reveals whether the patient died within the time of follow-up. The follow-up time in the study was at least one year for all patients.

We considered the following 16 variables as possible prognostic and predictive markers (predictors). These included 9 factor predictors: sex (male, female), histology (adenocarcinoma, squamous cell carcinoma), smoking status (ever smoked, never smoked), metformin use (yes, no), steroids use (yes, no), antibiotics use (yes, no), non-steroidal anti-inflammatory drugs use (NSAR) (yes, no), PD-L1 expression in the tumor cells (PD-L1TC) (not available (NA), <1%, 1–50%, >50%) and treatment (ICI, ICI, + Chemo). The remaining 7 predictors were continuous: body mass index (BMI) (kg/m^2^), lymphocytes (measured as 10^9^ cells per liter (G/l)), neutrophils (G/l), monocytes (G/l), basophils (0.01 G/l), eosinophils (0.01 G/l), and age at the start of ICI therapy (yrs). We note that the units of basophils and eosinophils were given in 0.01 G/l instead of 1 G/l, as the range of these predictors often did not exceed 1 G/l. The predictors antibiotics and steroids were excluded from the analysis related to irAE because these medications were given to treat the irAE.

At baseline, PD-L1 expression was evaluated on FFPE tumor sections using the E1L3N clone and scored if at least 50 tumor cells were available for analysis. Smoking status, age, BMI, and histology were determined at the start of therapy. Immune cell concentrations in the blood (lymphocytes, neutrophils, monocytes, basophils, and eosinophils) were measured at the day of treatment start. The administration of co-medications (metformin, steroids, antibiotics, and NSAR) was reported as “yes” if the medication was taken within one month before the start of IT and two months after the start of IT. For the statistical analyses, we excluded patients with undetermined histological subtypes and/or ongoing infections. Patients with missing values were excluded as well (*n* = 23). An exception was made for patients only missing the predictor PD-L1TC (*n* = 20), for which we created an additional category “NA”. The resulting number of first-line patients was *n* = 71 and the number of further-line patients was *n* = 111. The missingness plots of the initial data of both patient groups can be found in Figure S1. All statistical tests were carried out at significance level α=0.05 using the statistical software R, version 3.5.1.

### 2.2. Comparison of First- and Further-Line Patient Groups

We summarized the patient characteristics for the first- and further-line patient groups separately. We also computed separate Kaplan–Meier survival curves and conducted the corresponding log-rank test, comparing the survival of the two patient groups (Figure S2).

### 2.3. Pairwise Associations between Predictors and Responses

Before conducting the main statistical analyses, we assessed the pairwise associations between the predictors in both patient groups separately. For two continuous predictors, we used Spearman correlation (lies in [−1,1]); for two discrete predictors, we used Cramers’ V, which is based on a Chi-squared statistic (lies in [0,1]); and for a pair consisting of a discrete and a continuous predictor, we regressed the continuous predictor on the discrete predictor and took the square root of the explained variance (R^2^) multiplied by the sign of the regression coefficient (lies in [−1,1]).

In addition, we studied the pairwise associations between the three responses. We fitted a Cox proportional hazards model of overall survival on each binary response (Response3mt and irAE) and assessed the association between the two binary responses with a Fisher test.

### 2.4. Prognostic Markers for Binary Responses (Response3mt and irAE)

First, we ran univariate logistic regressions of the respective response on each predictor (including an intercept), and reported the following quantities: an estimate of the odds ratio (OR) for a one-unit increase in the predictor (or switch to another level from the reference level for factorial predictors), including 95% confidence intervals (CIs); the raw *p*-value for the predictor (using the Fisher test if the predictor was binary and the likelihood ratio test (LR test) if the predictor was not) and the multiple-testing adjusted *p*-value (Holm method, using 17 tests for Response3mt (two levels for PD-L1TC); and 15 tests for irAE (excluding steroids and antibiotics)) [16]. We assessed the sensitivity of the results with respect to outliers, by investigating how results changed if we removed influential observations. Here, influential observations were defined as having a Cook’s distance that exceeded 4/*n*, where *n* is the number of observations.

Second, we ran two-sample Wilcoxon tests for each continuous predictor, where the two groups were defined by the binary response. We reported the resulting *p*-value and the multiple-testing adjusted *p*-value (Holm method, using 7 tests because there are 7 continuous predictors). The Wilcoxon test is robust against outliers.

Last, we fitted a multivariate classification random forest, using the R-package *ranger* [17,18]. For each predictor, we reported the *p*-value of the impurity variable importance of a permutation test with 15,000 permutations. Again, we reported the multiple-testing adjusted *p*-value (Holm method, using 16 tests for Response3mt and 14 tests for irAE (PD-L1TC as one factor predictor)).

### 2.5. Prognostic Markers for Overall Survival

Similar to the binary responses, the main analyses conducted for the overall survival analysis were the following. First, we ran univariate Cox proportional hazard regressions of overall survival on each predictor (including an intercept), using the R-package *survival* [19]. This allowed us to report the following quantities: an estimate of the hazard ratio (HR) for a one-unit increase in the predictor including its 95% CI, the raw *p*-value for the predictor (LR-test), and the multiple testing adjusted *p*-value (Holm method, using 17 tests for Response3mt and 15 tests for irAE). In each Cox proportional hazard regression, we checked the proportional hazards assumption. We evaluated the sensitivity of the univariate Cox proportional hazard regression results with respect to removing influential observations. Here, influential observations were defined based on “dfbetas”, which are the estimated changes in the coefficients divided by their standard errors. We considered an observation to be influential if its absolute dfbetas value was greater than 0.3.

Second, we fitted a multivariate survival random forest, using the R-package *ranger*. For each predictor, we reported the *p*-value of the impurity variable importance of a permutation test with 15,000 permutations. Again, we reported the multiple-testing adjusted *p*-value (Holm method, using 16 tests for Response3mt and 14 tests for irAE (PD-L1TC as one factor predictor)).

Finally, we summarized the results of the univariate logistic and Cox regressions by plotting the log-OR and log-HR, including the respective CIs for each predictor, after standardizing the continuous predictors (centered to mean zero, scaled to variance one). This summary is suitable to compare effect sizes of different predictors.

### 2.6. Cut-Off Estimation for Continuous Predictors

In clinical studies, it is often of interest to divide the patients into treatment groups according to a predictor. These groups are usually determined based on a threshold t: one group consists of patients with predictor values ≤ t and the other group consists of patients with predictor values > t.

In both analyses, i.e., binary events and overall survival, we estimated these cut-offs with respect to the univariate logistic and univariate Cox regressions, using the R-package *maxstat* [20]. We reported the estimated cut-off and the *p*-value that corresponds to testing the null hypothesis that the distribution of the response is the same in both groups. In addition, for comparison and the sake of completeness, we binarized those continuous predictors that have a medically accepted healthy and corresponding non-healthy range and reported the *p*-values of the refitted models.

**Figure 1:**
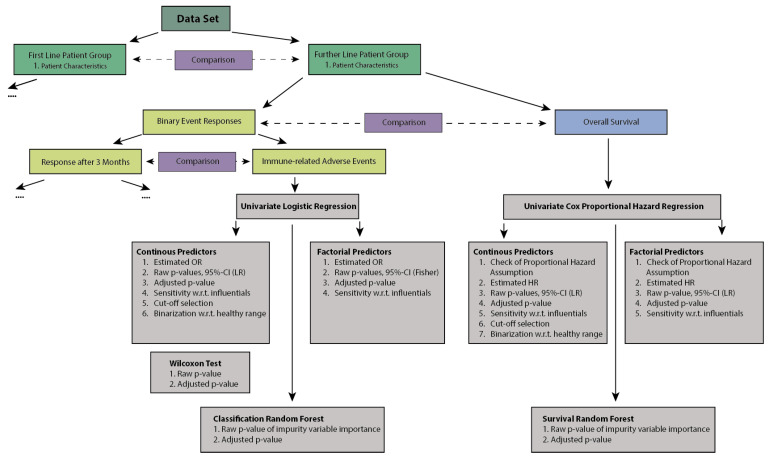
Schematic of statistical methods used.

All methods with corresponding context and explanation are summarized in Table S1. A diagram of the methods used in our analyses is displayed in Figure 1.

## 3. Results

### 3.1. Comparison of First- and Further-Line Patient Groups

We investigated stage-IV NSCLC patients who were treated with ICIs, either as first-line (*n* = 71) or further-line (*n* = 111) treatment at the University Hospital Zurich between 2015 and 2019. Patient characteristics of both groups are presented in Table 1. During the data collection time, 26 patients (37%) died in the first-line group, and 81 patients (73%) died in the further-line group. The median age at diagnosis was 67 years in the first-line patient group and 65 years in the further-line patient group. In both patient groups, approximately one third of the patients were female. First-line patients showed a statistically better median overall survival compared to the further-line patients (*p*-value of the log-rank test of < 0.0001) (Figure S2).

### 3.2. Pairwise Associations between Predictors and Responses

The associations between pairs of predictors within the two patient groups were all below 0.6 (Figure S3) and did not indicate severe multi-collinearity. The highest correlation of 0.57 was found in the first-line patient group between PD-L1TC and treatment choice. This can likely be explained by the fact that treatment was partially decided based on PD-L1TC expression. Other correlations in the first-line patient group with an absolute value above 0.4 were found between lymphocytes and eosinophils (0.47), and lymphocytes and monocytes (0.45). In the further-line patient group, the largest pairwise correlations were found between basophils and eosinophils (0.45), and between steroids and antibiotics (0.41).

The pairwise associations between the three responses (Response3mt, irAE, and overall survival) were also assessed for both patient groups. First, we fitted univariate Cox proportional hazard regressions of overall survival on Response3mt and irAE. First-line patients showed significantly prolonged survival if they responded within three months (*p* = 0.0132; HR: 0.3652; CI: 0.1586, 0.8407) (Figure S4C), but no significant association between survival and irAE development could be detected (*p* = 0.8966; HR: 0.9478; CI: 0.4213, 2.1324) (Figure S4D). Further-line patients also had a prolonged survival in case they responded within three months (*p* = 1.83 × 10^−7^, HR: 0.2657, CI: 0.1528, 0.4623) (Figure S4A) or if they developed an irAE (*p* = 0.00029, HR: 0.3859, CI: 0.2199, 0.6773) (Figure S4B). The relationship of the two binary responses, irAE and Response3mt, was analyzed with a Fisher test. They appeared to be significantly associated (*p* = 0.041) in the further-line patient group but not in the first-line patient group (*p* = 1) (Table S2).

### 3.3. Prognostic Markers for Binary Responses (Response3mt and irAE)

In the first-line patient group, no predictor was significantly associated with Response3mt with respect to any model. Similarly, we did not find any significant predictors for irAE in the univariate logistic regressions nor in the Wilcoxon tests. In the multivariate classification random forest, however, monocytes showed to be weakly significantly associated with the development of an irAE (*p* = 0.0235, adjusted *p*-value not significant) (Table 2; Tables S5 and S6).

In the further-line patient group, the univariate logistic regressions of Response3mt on each predictor yielded significant *p*-values for basophils, eosinophils, and steroids (*p* = 0.0027, *p* = 0.0215, and *p* = 0.0438, respectively). Increased basophil (OR: 1.3258; CI: 1.1017, 1.6152) and eosinophil counts (OR: 1.0227, CI: 1.002, 1.0446) as well as steroid intake (OR: 2.3571, CI: 1.0547, 5.4146) were associated with increased odds of responding within the first three months (Table 2, Table S3). The *p*-value for basophils even remained significant after multiple testing correction. Basophils and eosinophils were also significant in the Wilcoxon test (*p* = 0.0014 and *p* = 0.0446, respectively), and the *p*-value of basophils remained significant after multiple-testing correction. Only basophils showed a significant result in the multivariate classification random forest (*p* = 0.0177), fitted on all 16 predictors (not significant after multiple testing correction).

In the further-line patient group, the univariate logistic regressions of irAE on each predictor also found basophils to be significant (*p* = 0.0049), where increased basophil counts were associated with increased odds of developing an irAE (OR: 1.3108, CI: 1.0849, 1.6008) (Table 2, Table S4). However, this *p*-value ceased to be significant after multiple-testing correction. The basophil counts were also significant in the Wilcoxon test (*p* = 0.0112) and in the multivariate classification random forest (*p* = 0.004); however, both *p*-values were not significant after multiple testing correction. Thus, for further-line patients, the results suggest that basophil counts appear to be prognostic for Response3mt and for irAE, both marginally and when given all the other predictors.

Next, we were interested in the sensitivity of the univariate logistic regression results with respect to outliers. For all the tests where the *p*-value was significant before and/or after removing influential observations, Table S7 shows both *p*-values. Interestingly, the *p*-values only got more significant after removing influential observations. Basophil counts now remained significant in the further-line patient group even when adjusting for multiple testing for Response3mt (new raw *p* = 0.00033, new adjusted *p* = 0.0056) as well as for irAE (new raw *p* = 0.00023, new adjusted *p* = 0.0038).

### 3.4. Prognostic Markers for Overall Survival

We checked the proportional hazards assumption for each univariate Cox proportional hazards model. We found a significant violation only for basophil counts in the first-line patient group (*p* = 0.005) and decided to still use the model in this case.

In the first-line patient group, the univariate Cox proportional hazard regressions yielded significant *p*-values for lymphocytes, histology, NSAR, and treatment (*p* = 0.0031, *p* = 0.0239, *p* = 0.0078, and *p* = 0.042, respectively). None of these *p*-values remained significant after adjusting for multiple testing. Increased lymphocyte counts were associated with decreased hazards of dying (HR: 0.3912, CI: 0.1984, 0.7714). The hazards of dying were also decreased for the histology of adenocarcinoma compared to squamous cell carcinoma (HR: 0.3273; CI: 0.135, 0.7934) and for the intake of NSAR (HR: 0.2478; CI: 0.0738, 0.8325). In the multivariate survival random forest, histology, neutrophils and NSAR were significant (*p* = 0.0202, *p* = 0.0088, and *p* = 0.0336, respectively), while ICI & Chemo did not change the risk of dying compared to ICI alone (Table 3, Table S9). None of these *p*-values remained significant after adjusting for multiple testing. Here, neutrophils showed the smallest *p*-value, whereas they were not significant in the univariate Cox regression model.

In the further-line patient group, the univariate Cox proportional hazard regressions yielded significant *p*-values for lymphocytes, PD-L1TC level > 50%, and smoking (*p* = 0.0475, *p* = 0.0101, and *p* = 0.0441, respectively) (Table 3, Table S8). Increased lymphocyte counts (HR: 0.7268; CI: 0.524, 1.008), higher PD-L1 expression in tumor cells (HR: 0.3579; CI: 0.1494, 0.8575), and a positive smoking status (HR: 0.4883; CI: 0.2571, 0.9274) were all associated with decreased hazards of dying. All of these *p*-values became non-significant after adjusting for multiple testing. In the multivariate survival random forest, we found (weakly) significant *p*-values only for monocytes and smoking (*p* = 0.0453 and *p* = 0.049, respectively), which were not significant after adjusting for multiple testing.

We again assessed the sensitivity of our results with respect to the removal of influential observations. As before, we found that removing influential observations only strengthened the univariate Cox regression results, with *p*-values becoming smaller. In the first-line patient group, the two predictors NSAR (new raw *p* = 0.00002, new adjusted *p* = 0.0004) and lymphocytes (new raw *p* = 0.001, new adjusted *p* = 0.0166) now remained significant even when adjusting for multiple testing.

**Figure 2:**
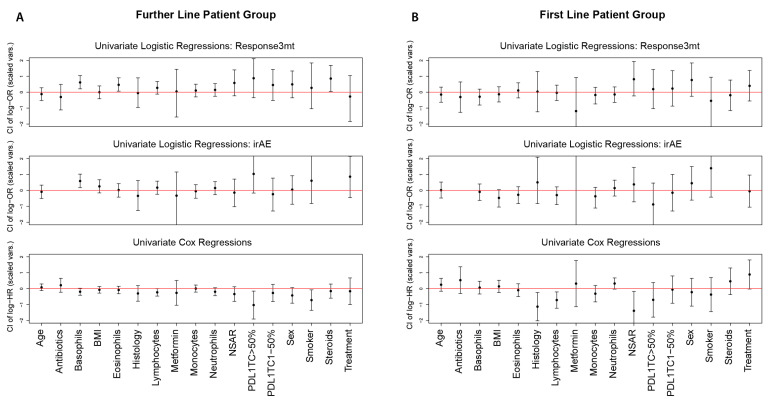
Estimated log-OR (log-HR) and 95% CI for each univariate logistic (Cox) regression using standardized predictors for (**A**) the further-line patient group and (**B**) the first-line patient group.

Figure 2 summarizes all findings, where we plotted the estimated log-ORs from the univariate logistic regressions and the estimated log-HRs from the univariate Cox proportional hazard regressions, after standardizing the continuous predictors. This figure can be used to assess whether effect sizes are clinically relevant and to compare effect sizes for different predictors, as the unit for a change in log-OR or log-HR is now the standard deviation of each predictor. It seemed that, for all significant results, the effect size is potentially relevant for clinical practice.

### 3.5. Cut-Off Estimation for Continuous Predictors

We estimated cut-off values for continuous predictors and tested if the resulting subgroups behaved differently with respect to the binary Response3mt or irAE and overall survival.

In the first-line patient group, we found a significant cut-off of 0.51 G/l (*p* = 0.0266) in monocyte counts considering irAE, where high monocyte counts were associated with increased odds of developing an irAE. Considering overall survival, we found the cut-off of 1.78 G/l in lymphocyte counts to be significant (*p* = 0.029), where high lymphocyte counts were associated with decreased odds of dying. The only significant result we found in the healthy range analysis was the one of neutrophils (*p* = 0.0346), where neutrophil counts in the healthy range were associated with increased odds of developing an irAE (Tables S11 and S12).

In the further-line patient group, we found a significant cut-off of 0.02 G/l (*p* = 0.004) in basophil counts considering Response3mt and a significant cut-off of 0.05 G/l (*p* = 0.013) considering irAE. High basophil counts were associated with increased odds of responding within three months and developing an irAE. The only significant result we found in the healthy range analysis is the one of lymphocyte counts, where lymphocyte counts in the healthy range were associated with increased odds of responding within three months (*p* = 0.0466) and decreased hazard of dying (*p* = 0.0415) (Tables S11 and S12).

## 4. Discussion

The implementation of ICI therapies targeting the PD-1/PD-L1 axis has revolutionized the treatment of metastatic NSCLC and improved overall survival rates in these difficult-to-treat patients. However, patients can suffer from heavy side effects and only a subgroup of patients benefits from the treatment with ICIs. Thus, it is crucial to find (bio-)markers of response, to aid selection of patients that will likely benefit from the treatment and to stratify patients in future clinical trials.

Many studies have discussed clinical markers as prognostic factors for NSCLC patients treated with ICI therapy (reviewed in [21]). However, proper statistical analyses are often missing, and issues, such multiple testing and post-selection inference (described below), tend to be overlooked.

In this study, we used retrospectively collected clinical baseline markers of two cohorts of patients with stage-IV NSCLC, treated in either a first-line or further-line therapy setting. The primary aim of this study was to find predictive and prognostic markers for response to ICIs, quantified by tumor size reduction within the first three months of treatment (Response3mt), development of immune-related adverse events (irAE), and overall survival, where the analyses included robustness checks of the results.

In order to find prognostic markers for the binary Response3mt and development of irAE, we performed univariate logistic regressions for each predictor assuming a linear relation between each predictor and the log-odds of the response. The robust Wilcoxon test was used as an additional univariate test that does not rely on any parametric assumption. In addition, we analyzed our data using the classification random forest, which is both multivariate and nonparametric, providing yet another perspective. Similarly, for the overall survival analysis, we performed univariate Cox proportional hazard regressions, assuming a linear relationship between each predictor and the log-hazard, resulting in the well-known proportional hazards assumption. In accordance with the binary response analyses, we also analyzed our data using a survival random forest, which is again both multivariate and nonparametric.

In the multivariate analyses, we did not conduct the often-used best sub-model selection because it is problematic. First, the definition of the best sub-model is quite arbitrary, due to the fact that a few models often have a very similar performance. Second, the *p*-values of the best sub-model are biased (they tend to be too small), if the sub-model is fitted on the same data on which it was selected. The latter is known as the problem of post-selection inference. Another problem is the one of multiple testing. The probability of incorrectly rejecting a null-hypothesis of no effect increases with the number of tests. Therefore, proper inflation of the *p*-values is required to get guarantees on the rate of wrongly rejected null hypotheses [22]. We performed adjustment of the *p*-values with the Holm method to correct for this multiple testing problem. Often, this is not performed in studies analyzing clinical data sets with many tests, leading to overly optimistic findings and wrong conclusions. Finally, we also checked whether the model assumptions were met, and we checked the sensitivity of our results with respect to the removal of influential observations or outliers.

Overall, we obtained different results for the first-line (*n* = 71) and further-line (*n* = 111) patient group, indicating that these groups may be fundamentally different. For example, first-line patients had a better overall survival than further-line patients. Further-line patients had a prolonged survival in case they responded within three months or developed an irAE, whereas first-line patients only showed a positive association between responding within three months and survival. Several studies reported association of irAE development and longer overall survival in late-stage NSCLC patients [23,24,25].

**Figure 3:**
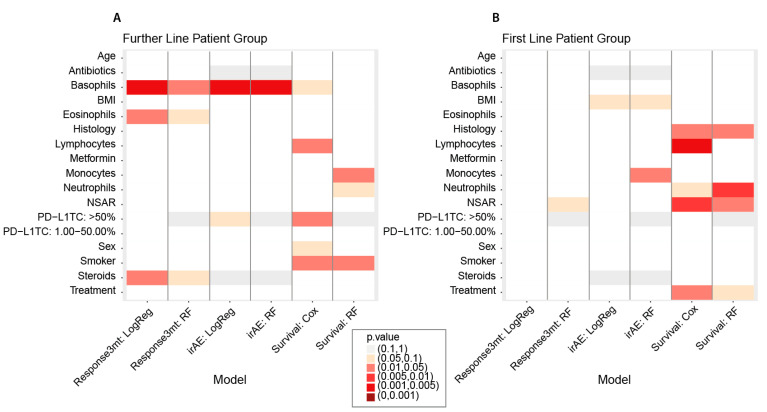
Summary plot of significance of *p*-values of each predictor in each of the analyses for (**A**) the further-line patient group and (**B**) the first-line patient group. LogReg: logistic regression; RF: random forest; Cox: Cox proportional hazard regression.

In the further-line patient group, the most significant result was found for blood basophil counts at baseline for Response3mt and irAE in all the respective tests, where the effect size was potentially relevant (Figure 3). High basophil counts were associated with increased odds of responding within three months and developing an irAE. This is in line with our cut-off finding that patients with basophil count values higher than 0.02 G/l (0.05 G/l) had increased odds of responding within three months (and of developing an irAE, respectively). If influential observations were removed (three for Response3mt and four for irAE), the new *p*-values of the logistic regressions became even more significant. An increase in basophil counts was borderline not significant in the univariate Cox regression, but the effect pointed in the same direction of a treatment benefit. The contribution of basophils to anti-tumor immunity in the context of ICI therapy is currently unclear. Basophils express cytotoxic molecules, such as granzyme b, and can have cytotoxic effects on tumor cells. Several studies described high basophil counts to be favorable for the outcome. In an ovarian cancer cohort consisting of 53 patients, patients with high basophil counts had a better overall survival compared to patients with low counts, independently of the treatment [26]. Furthermore, in colorectal cancer, low basophil counts were associated with poor prognosis [27,28]. Similar results were obtained in melanoma patients treated with ICI therapy, where high basophil counts were positively correlated with a positive outcome [29]. In our study, we found that high basophil counts may be beneficial for ICI treatment response in further-line patients. This hypothesis has to be tested on independent data.

Furthermore, we found that in the further-line patient group an increase in eosinophil counts significantly increased the odds of responding within three months with a potentially relevant effect size. After multiple testing correction, however, the *p*-value was no longer significant. There was one influential observation, and removing it resulted in a smaller *p*-value. High eosinophil counts have already been shown to be predictive for response in other studies analyzing advanced NSCLC patients. Nevertheless, this statistical analysis performed the best subset selection and reported *p*-values computed on the same data [30,31].

We also found that a PD-L1TC level of >50% was favorable for overall survival. This is in line with other studies, which found that high baseline PD-L1 levels were favorable for survival [32,33,34,35].

In the first-line patient group we obtained more diffuse results. For Response3mt, we found no significant result for any of the predictors, but we found that patients with neutrophil counts in the healthy range responded significantly better within three months. With respect to irAE, we found a significant cut-off of 0.51 for monocytes, where higher monocyte counts corresponded to an increased odds of developing an irAE. Increased baseline monocyte counts were already associated with the development of irAE in other studies, but also with weak *p*-values [36]. We also found monocytes to be significant with respect to the survival random forest for irAE (Figure 3).

Concerning overall survival, we found that in the first-line patient group the histology of adenocarcinoma was beneficial compared to squamous cell carcinoma. In addition, higher lymphocyte counts and an intake of NSAR indicated a decrease in the hazard of dying. We also found a significant cut-off of 1.78 for lymphocytes, were patients with counts above this cut-off tended to have better survival. Histology and NSAR were also significant with respect to the survival random forest. The significance of the *p*-values of histology, lymphocytes, and NSAR increased when removing influential observations, and the ones of lymphocytes and NSAR now even stayed significant after *p*-value adjustment, indicating their potential as a predictor for overall survival. The histology of adenocarcinoma and high lymphocyte counts were already described to have a favorable influence on survival [32,33,34,35]. Lung cancer patients with longer overall survival had enhanced numbers of CD3, CD4, and CD8 T cell in the blood at baseline [37], and high lymphocyte counts were associated with better overall survival in recurrent NSCLC patients [38,39]. Concerning the advantage or disadvantage of treatment with NSAR, there are currently no conclusive results. Certain studies describe similar outcomes in NSCLC patients [40], while other studies could not find an effect of NSAR intake on improved survival in melanoma patients [41]. A positive influence of NSAR intake was described on the number of tumor-infiltrated CD4 and CD8 T cells in a mouse model of breast cancer [42] and on reducing proliferation of suppressive myeloid cells [43]. Interestingly, COX-2 inhibition led to intra-tumoral expression of IFN-γ and increased the intra-tumoral accumulation of effector T cells [44]. Thus, the positive effect of NSAR intake needs to be further investigated.

There are several limitations of this study that restrict the generalizability of the results. The sample sizes of the two patient groups were only moderate, and the data were collected in only one hospital in Switzerland, which might induce a bias. This study is exploratory, and the results must be seen as hypotheses that have to be verified on independent data, preferably in a prospective fashion. In addition, in several of our analyses we found only weakly significant *p*-values close to 0.05 before multiple-testing adjustment. Therefore, we did not discuss these results any further and they would have to be validated in independent cohorts.

## 5. Conclusions

We analyzed data from two cohorts of stage-IV NSCLC patients treated with ICIs that were collected during normal clinical routine, with the aim of finding biomarkers for treatment response. We performed a comprehensive statistical analysis and discussed statistical problems occurring in many biomarker studies previously published. We found several significant results, suggesting possible (bio-)markers for treatment response, which need to be validated in independent and larger cohorts. These (bio-)markers could be of help as predictors of ICI therapy response or for patient stratification in clinical trials. Our strongest result suggests that an increase in basophil counts may be beneficial for treatment response in further-line patients.

## Figures and Tables

**Table 1:** Patient characteristics.

Total		Further-Line Patient Group	First-Line Patient Group
		*n* = 111	*n* = 71
Age (yrs)	median (range)	65 (36–89)	67 (43–82)
	standard deviation	9.7	9.3
Sex	female	35 (31.5%)	22 (31%)
	male	76 (68.5%)	49 (69%)
Smoker	yes	100 (90.1%)	63 (88.7%)
	no	11 (9.9%)	8 (11.3%)
BMI	median (range)	24 (17.1–42.5)	24.5 (16.2–35.2)
Histologic subtype	Adenocarcinoma	84 (75.7%)	59 (83.1%)
	Squamous cell carcinoma	27 (24.3%)	12 (16.9%)
Immune-rel. Adverse Events	yes	31 (27.9%)	24 (33.8%)
	no	80 (72.1%)	47 (66.2%)
Lymphocytes (G/l)	median	1.09 (0.17–3.75)	1.47 (0.31–4.7)
	standard deviation	0.7183	0.7579
	Normal range (1.5–4)	34 (30.6%)	34 (47.9%)
	Not in normal range	77 (69.4%)	37 (52.1%)
Monocytes (G/l)	median	0.81 (0.09–1.98)	0.69 (0.02–3.1)
	standard deviation	0.3308	0.4058
	Normal range (0.16–0.95)	80 (72.1%)	54 (76.1%)
	Not in normal range	31 (27.9%)	17 (23.9%)
Neutrophils (G/l)	median	4.76 (0.3–13.89)	5.1 (1.25–16.7)
	standard deviation	2.598	2.7834
	normal range (1.4–8)	95 (85.6%)	58 (81.7%)
	Not in normal range	16 (14.14%)	13 (18.3%)
Eosinophils (G/l)	median	0.12 (0–1.09)	0.16 (0.01–0.5)
	standard deviation	0.2069	0.1295
	Normal range (0–0.7)	107 (96.4%)	71 (100%)
	Not in normal range	4 (3.6%)	0 (0%)
Basophils (G/l)	median	0.02 (0.01–0.1)	0.04 (0.01–0.12)
	standard deviation	0.0217	0.0223
	Normal range (0–0.15)	111 (100%)	71 (100%)
	Not in normal range	0 (0%)	0 (0%)
PDL1-TC	<1%	45 (40.5%)	23 (32.4%)
	1–50%	33 (29.8%)	28 (39.4%)
	>50%	15 (13.5%)	19 (26.8%)
	not available	18 (16.2%)	1 (1.4%)
Response (CR, PR) at 3 months	yes	36 (32.4%)	36 (50.7%)
	no	75 (67.6%)	35 (49.3%)
Antibiotics	yes	55 (49.5%)	42 (59.2%)
	no	56 (50.5%)	29 (40.8%)
NSAR	yes	42 (37.8%)	20 (28.2%)
	no	69 (62.2%)	51 (71.8%)
Steroids	yes	52 (46.8%)	43 (60.6%)
	no	59 (53.2%)	28 (39.4%)
Metformin	yes	9 (8.1%)	4 (5.6%)
	no	102 (91.9%)	67 (94.4%)
Treatment	ICI & Chemo	11 (9.9%)	42 (59.2%)
	ICI	100 (90.1%)	29 (40.8%)
OS (days)	median (range)	340 (9–1807)	461 (61–1198)
	standard deviation	463.3	290.2

OS: overall survival is calculated from the day of start of treatment to death (or censored); NSAR: non-steroidal anti-rheumatic drug; CR: complete response; PR: partial response; SD: stable disease; PD: progressing disease; BMI: body mass index; PD-L1TC: PD-L1 expression in tumor cells.

**Table 2:** Significant results for response at three months and development of immune-related adverse events for the first- and further-line patient groups (complete list of results is presented in Tables S3–S6).

			Univariate Methods						Multivariate Methods	
			Univariate Logistic Regressions				Additional Tests		Classification Random Forest	
Patient Group	Response Variable	Predictor	Estimate (OR)	95% CI	Raw *p*-Value (LR/Fisher)	Adjusted *p*-Value (Holm)	Raw *p*-Value (Wilcoxon)	Adjusted *p*-Value (Holm)	Raw Impurity Importance *p*-Value (RF)	Adjusted *p*-Value (Holm)
Further Line	Response3mt	Basophils (0.01 G/l)	1.3258	(1.1017,1.6152)	0.0027	0.0453	0.0014	0.001	0.0177	0.2832
		Eosinophils (0.01 G/l)	1.0227	(1.0032,1.0446)	0.0215	0.3443	0.0446	0.2679	0.0978	1
		Steroids: TRUE	2.3571	(1.0547,5.4146)	0.0438	0.6564	-	-	0.0515	0.7725
	irAE	Basophils (0.01 G/l)	1.3108	(1.0849,1.6008)	0.0049	0.0839	0.0112	0.0781	0.0040	0.056
First Line	irAE	Monocytes (1 G/l)	0.4011	(0.0652,1.5982)	0.2167	1	0.1849	1	0.0235	0.329

OR: odds ratio; LR: likelihood ratio; RF: random forest.

**Table 3:** Significant results for overall survival for the first- and further-line patient groups (complete list of results is presented in Tables S8 and S9).

		Univariate Methods				Multivariate Methods	
		Univariate Cox Proportional Hazard Regressions				Survival Random Forest	
Patient Group	Predictor	Estimate (HR)	95% CI	Raw *p*-Value (LR)	Adjusted *p*-Value (Holm)	Raw Impurity Importance *p*-Value (RF)	Adjusted *p*-Value (Holm)
Further Line	Lymphocytes (G/l)	0.7268	(0.524, 1.008)	0.0475	0.7119	0.6327	1
	PD-L1TC: >50%	0.3579	(0.1494, 0.8575)	0.0101	0.1717	0.2144 (for whole predictor PD-L1TC)	1
	Smoker: TRUE	0.4883	(0.2571, 0.9274)	0.0441	1	0.0490	0.7350
	Monocytes (G/l)	1.0227	(0.5245, 1.9944)	0.9474	1	0.0453	0.728
First Line	Histology: Adenocarcinoma	0.3273	(0.135, 0.7934)	0.0239	0.3579	0.0202	0.303
	Lymphocytes (G/l)	0.3912	(0.1984,0.7714)	0.0031	0.0535	0.1721	1
	NSAR: TRUE	0.2478	(0.0738, 0.8325)	0.0078	0.1256	0.0336	0.4708
	Treatment: ICI & Chemo	2.4608	(0.9909, 6.1112)	0.0420	0.5886	0.0989	1
	Neutrophils (G/l)	1.1255	(0.9943, 1.274)	0.081	1	0.0088	0.1408

OR: odds ratio; LR: likelihood ratio; RF: random forest.

## Data Availability

The data are not publicly available due to legal and privacy issues.

## Supplementary Materials

The following are available online at https://www.mdpi.com/article/10.3390/cancers14010075/s1. Figure S1: Missingness plots of the further-line patient group (A) and first-line patient group (B). Figure S2: Kaplan–Meier Curves of the first-line patient group (blue) and the further-line patient group (red) with the *p*-value of the corresponding log-rank test. Figure S3: Pairwise associations between all analyzed predictors (red: negative association, blue: positive association) in the further-line patient group (A) and first-line patient group (B). Figure S4: Kaplan–Meier curves of response within three months (Response3mt) and development of immune-related adverse events (irAE) in the further-line patient group (A,B) and the first-line patient group (C,D) with the p-value of the corresponding log-rank test. Table S1: Summary table of the statistical methods used. Table S2: Fisher test relating response within three months (Response3mt) and development of immune-related adverse events (irAE) in the further-line patient group and the first-line patient group. Table S3: Results of all analyses with respect to response within 3 months (Response3mt) in the further-line patient group. Table S4: Results of all analyses with respect to development of immune-related adverse events (irAE) in the further-line patient group. Table S5: Results of all analyses with respect to response within 3 months (Response3mt) in the first-line patient group. Table S6: Results of all analyses with respect to development of immune-related adverse events (irAE) in the first-line patient group. Table S7: Univariate logistic regressions on response within three months (Response3mt) and development of immune-related adverse events (irAE) with influential observations removed in the further-line patient group and the first-line patient group. Table S8: Results of all analyses with respect to overall survival in the further-line patient group. Table S9: Results of all analyses with respect to overall survival in the first-line patient group. Table S10: Univariate Cox proportional hazard regressions on overall survival with influential observations removed in the further-line patient group and the first-line patient group. Table S11: Significant cut-off values of continuous variables with respect to univariate logistic regressions on response within three months (Response3mt) and development of immune-related adverse events (irAE) in the further-line patient group and the first-line patient group. Table S12: Significant cut-off values of continuous variables with respect to univariate Cox regressions on overall survival in the further-line patient group and the first-line patient group.
