# Supplementary material for: Comprehensive Statistical Exploration of Prognostic (Bio-)Markers for Responses to Immune Checkpoint Inhibitor in Patients with Non-Small Cell Lung Cancer
Source: Cancers (Basel). 2021 Dec 24;14(1):75. doi: 10.3390/cancers14010075 (PMC8750624; doi:10.3390/cancers14010075)
Supplement: Supplementary file 1 [file cancers-14-00075-s001.zip › cancers-1487457-supplementary.pdf]

Supplementary Information

Figure S1: Missingness plots of the further-line patient group (A) and first-line patient group (B)

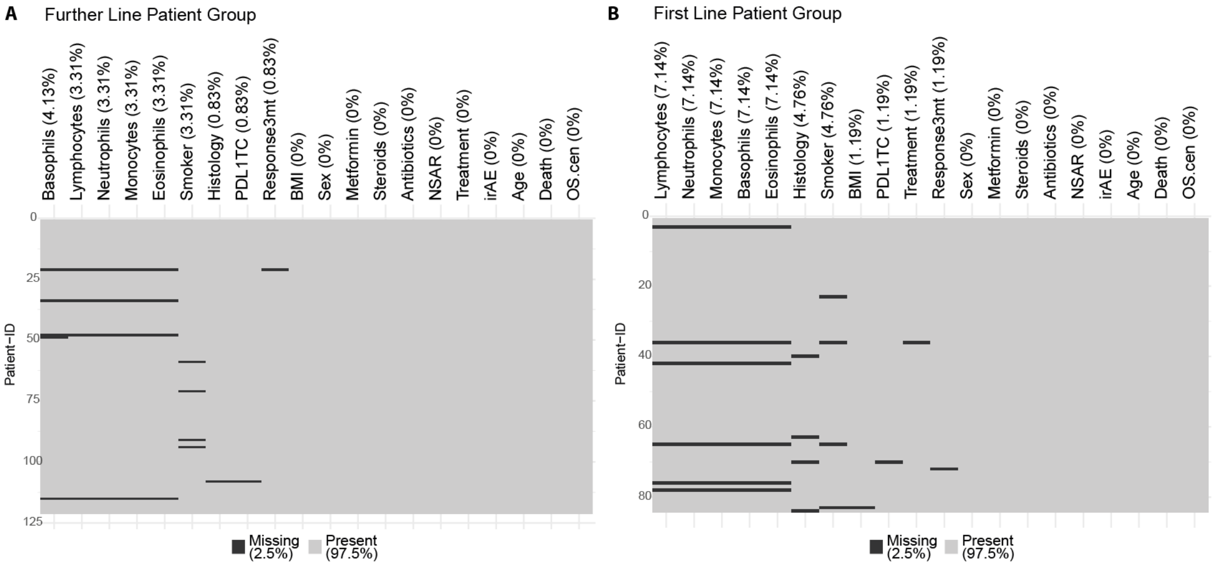

Figure S2: Kaplan-Meier Curves of the first-line patient group (blue) and the further-line patient group (red) with the p-value of the corresponding log-rank test

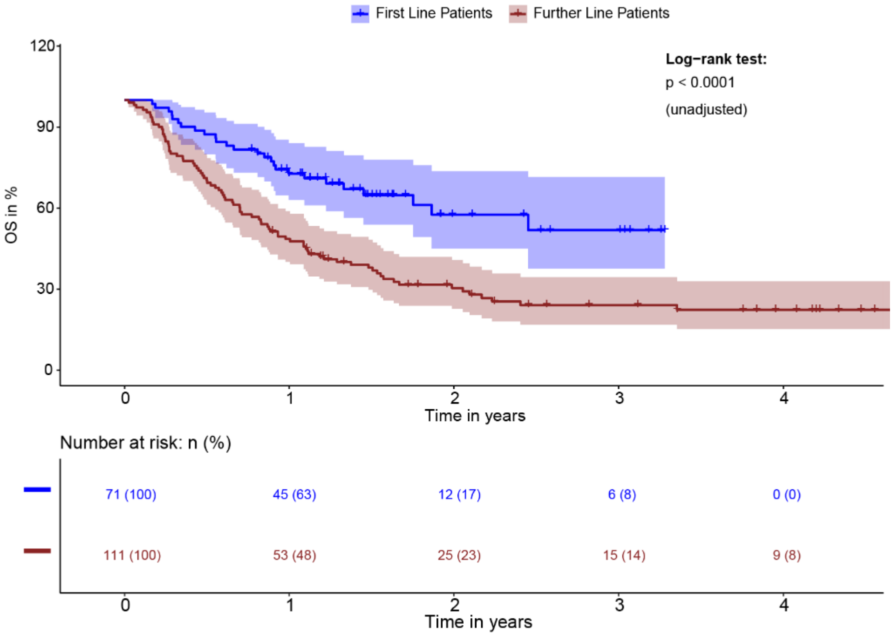

Figure S3: Pairwise associations between all analysed predictors (red: negative association, blue: positive association) in the further-line patient group (A) and first-line patient group (B)

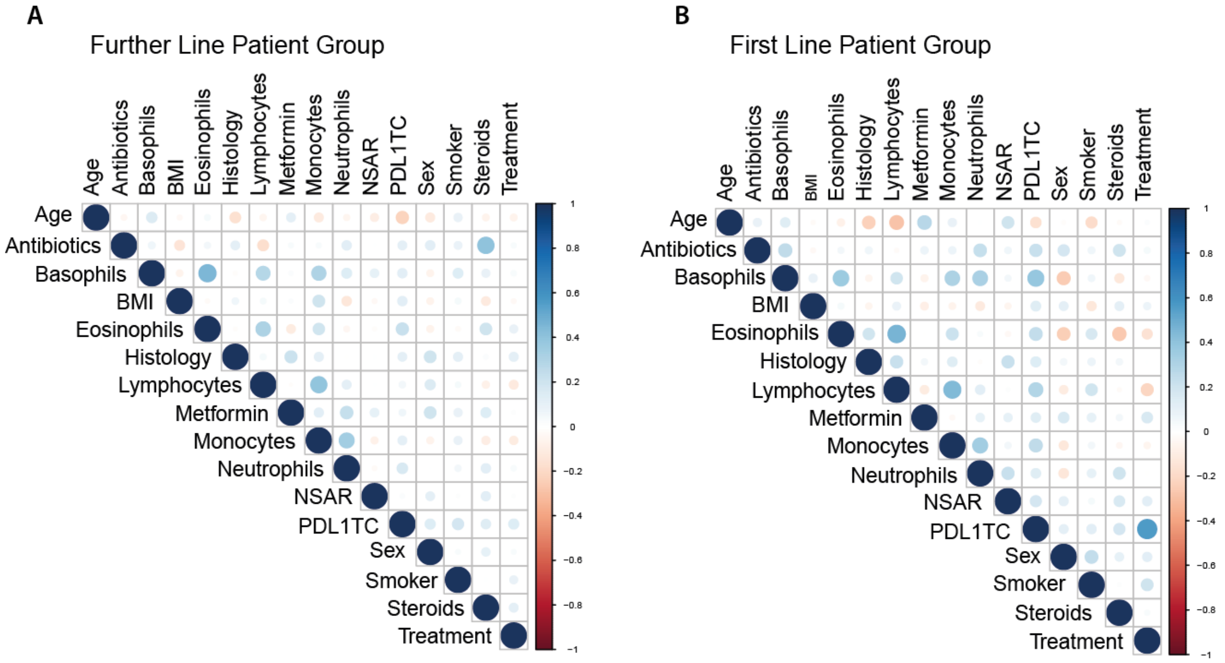

Figure S4: Kaplan-Meier curves of response within three months (Response3mt) and development of immune-related adverse events (irAE) in the further-line patient group (A,B) and the first-line patient group (C,D) with the p-value of the corresponding log-rank test.

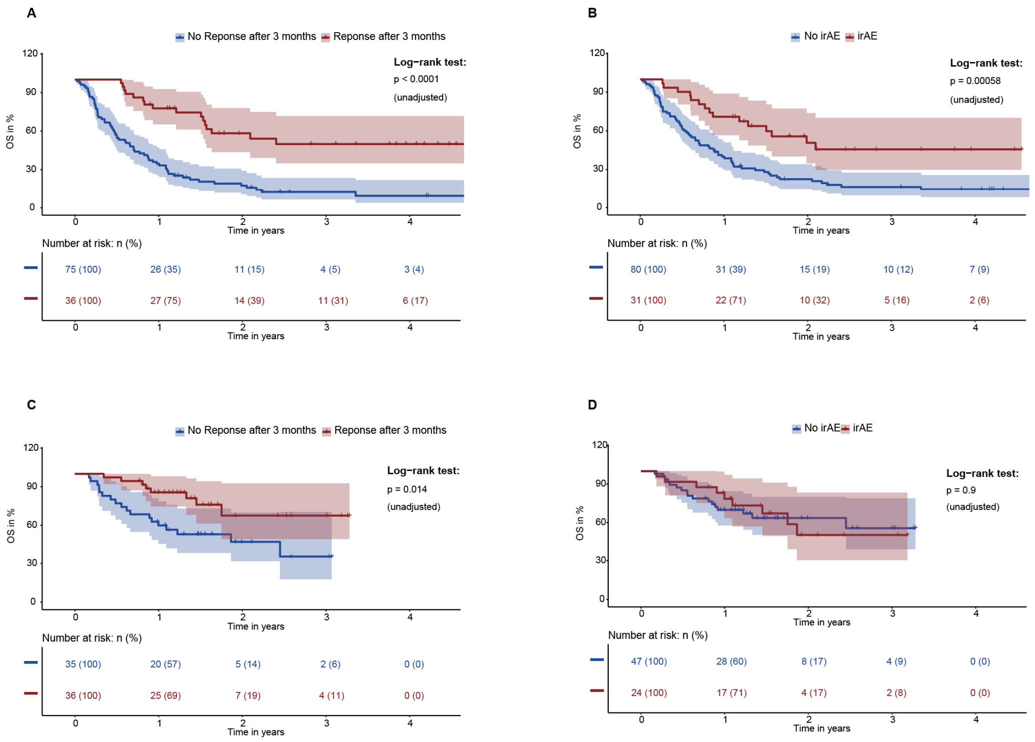

Table S1: Summary table of the statistical methods used.

Method	Context	Explanation
Univariate Logistic Regression	Applied to binary responses (Response3mt and irAE) and each univariate predictor	• Standard model for this data type assuming linear relation between predictor and log-odds of the response.<br>• Estimate of odds-ratio of responding vs not responding indicates the sign of the relationship (positive or negative) of predictor with response.<br>• With likelihood-ratio test (for continuous predictors) or Fisher test (for binary predictors) get a significance value.<br>• Applied to each combination of univariate predictor and binary response. Thus, these marginal results are to be understood independently of value of other predictors.
Wilcoxon Test	Applied to binary responses (Response3mt and irAE) and each univariate continuous predictor	• Standard test for relationship of univariate predictor and response.<br>• Does not rely on parametric assumption, in particular does not make linearity assumption, robust to outliers<br>• Applied to each combination of univariate continuous predictor and binary response.<br>• Aim of confirming the results of the univariate logistic regressions.
Classification Random Forest	Applied to binary responses (Response3mt and irAE) and all predictors	• Both multivariate and nonparametric, providing another perspective.<br>• An ensemble learner consisting of 500 classification trees.<br>• Used impurity variable importance measure, a weighted mean of the improvement in each tree in the splitting criterion.<br>• Reported the p-value of permutation test with 15'000 permutations considering the impurity variable importance.
Univariate Cox Proportional Hazard Regression	Applied to Overall Survival (a combination of indicator of death-indicator and survival time) and each univariate predictor	• Standard model for this data type assuming linear relation between predictor and log-hazard, resulting in well-known proportional hazards assumption.<br>• Estimate of hazard-ratio of dying vs not dying indicates the sign of the relationship (positive or negative) of predictor with response.<br>• With likelihood-ratio test get a significance value.<br>• Applied to each univariate predictor and overall survival. Thus, these marginal results are to be understood independently of value of other predictors.
Survival Random Forest	Applied to overall survival as response and all predictors	• A similar algorithm as classification random forest using overall survival as the response.<br>• See description above.

Table S2: Fisher-Test relating response within three months (Response3mt) and development of immune-related adverse events (irAE) in the further-line patient group and the first-line patient group.

		irAE		Raw p-Value	Estimated OR
Response3mt	Further Line	No	Yes	0.041	2.609
	No	59	16		
	yes	21	15		
	First Line	No	Yes	1	0.959
	No	23	12		
	yes	24	12		

OR: odds ratio

Table S3: Results of all analyses with respect to response within 3 months (Response3mt) in the further-line patient group.

	Univariate Methods						Multivariate Methods	
	Univariate Logistic Regression				Additional Tests		Classification Random Forest	
Variable	Estimate (OR)	95%-CI	Raw P-Value (LR/Fisher)	Adjusted P-Value (Holm)	Raw P-Value (Wilcoxon)	Adjusted P-value (Holm)	Raw Impurity Importance P-Value	Adjusted P-Value (Holm)
Age (years)	0.9877	(0.9474, 1.0293)	0.5545	1	0.7172	1	0.2249	1
Antibiotics: TRUE	0.7385	(0.3292, 1.6371)	0.5442	1	-	-	0.3241	1
Basophils (0.01 G/l)	1.3258	(1.1017, 1.6152)	0.0027	0.0453	0.0014	0.001	0.0177	0.2832
BMI (kg/m^2)	0.9997	(0.9131, 1.0908)	0.9946	1	0.8181	1	0.7567	1
Eosinophils (0.01 G/l)	1.0227	(1.0032, 1.0446)	0.0215	0.3443	0.0446	0.2679	0.0978	1
Histology: Adenocarcinoma	0.9474	(0.3829, 2.4618)	1	1	-	-	0.5600	1
Lymphocytes (G/l)	1.4628	(0.8446, 2.5584)	0.1734	1	0.0736	0.3679	0.5994	1
Metformin: TRUE	1.0455	(0.2105, 4.2258)	1	1	-	-	0.9363	1
Monocytes (G/l)	1.3763	(0.4078, 4.5868)	0.6012	1	0.3610	1	0.9731	1
Neutrophils (G/l)	1.0572	(0.9063, 1.2303)	0.4715	1	0.2814	1	0.8348	1
NSAR: TRUE	1.7895	(0.7933, 4.0513)	0.2098	1	-	-	0.2299	1
PD-L1TC: 1.00-50.00%*	1.5714	(0.5941, 4.1851)	0.3607	1	-	-	0.7428	1
PD-L1TC: >50%*	2.4062	(0.7075, 8.2055)	0.1573	1	-	-	-	-
Sex: Woman	1.6364	(0.7013, 3.7865)	0.2795	1	-	-	0.2617	1
Smoker: TRUE	1.3134	(0.3536, 6.2912)	1	1	-	-	0.9361	1
Steroids: TRUE	2.3571	(1.0547, 5.4146)	0.0438	0.6564	-	-	0.0515	0.7725
Treatment: ICI & Chemo	0.7614	(0.159, 2.8277)	1	1	-	-	0.9616	1

OR: odds ratio, LR: likelihood ratio

Table S4: Results of all analyses with respect to development of immune-related adverse events (irAE) in the further-line patient group.

	Univariate Methods						Multivariate Methods	
	Univariate Logistic Regressions				Additional Tests		Classification Random Forest	
Variable	Estimate (OR)	95%-CI	Raw P-Value (LR/Fisher)	Adjusted P-Value (Holm)	Raw P-Value (Wilcoxon)	Adjusted P-Value (Holm)	Raw Impurity Importance P-Value	Adjusted P-Value (Holm)
Age (years)	0.9910	(0.9491,1.0348)	0.6780	1	0.3892	1	0.4048	1
Basophils (0.01 G/l)	1.3108	(1.0849,1.6008)	0.0049	0.0839	0.0112	0.0781	0.0040	0.056
BMI (kg/m^2)	1.0574	(0.9659,1.1592)	0.2241	1	0.5475	1	0.4988	1
Eosinophils (0.01 G/l)	1.001	(0.9798,1.0207)	0.9175	1	0.4877	1	0.6933	1
Histology: Adenocarcinoma	0.7097	(0.2819,1.866)	0.4703	1	-	-	0.1554	1
Lymphocytes (G/l)	1.2747	(0.7154,2.2478)	0.4032	1	0.5541	1	0.4452	1
Metformin: TRUE	0.7192	(0.1031,3.1875)	1	1	-	-	0.7238	1
Monocytes (G/l)	0.8539	(0.2271,2.9776)	0.8071	1	0.8334	1	0.7421	1
Neutrophils (G/l)	1.0596	(0.9026,1.238)	0.4692	1	0.3965	1	0.7358	1
NSAR: TRUE	0.8694	(0.3584,2.0361)	0.8293	1	-	-	0.7957	1
PD-L1TC: 1.00-50.00%*	0.7877	(0.2738,2.1683)	0.6465	1	-	-	0.5703	1
PD-L1TC: >50%*	2.8132	(0.8475,9.651)	0.0907	1	-	-	-	-
Sex: Woman	1.0476	(0.4188,2.5143)	1	1	-	-	0.7955	1
Smoker: TRUE	1.8380	(0.4398,12.5404)	0.7247	1	-	-	0.6791	1
Treatment: ICI & Chemo	2.3718	(0.6364,8.5297)	0.2856	1	-	-	0.1115	1

OR: odds ratio, LR: likelihood ratio

Table S5: Results of all analyses with respect to response within 3 months (Response3mt) in the first-line patient group.

	Univariate Methods						Multivariate Methods	
	Univariate Logistic Regressions				Additional Tests		Classification Random Forest	
Variable	Estimate (OR)	95%-CI	Raw P-Value (LR/Fisher)	Adjusted P-Value (Holm)	Raw P-Value (Wilcoxon)	Adjusted P-Value (Holm)	Raw Impurity Importance P-Value	Adjusted P-Value (Holm)
Age (years)	0.9842	(0.9342,1.0353)	0.5365	1	0.5195	1	0.5194	1
Antibiotics: TRUE	0.7386	(0.2824,1.9063)	0.6312	1	-	-	0.6484	1
Eosinophils (0.01 G/l)	0.8807	(0.6962,1.0907)	0.2474	1	0.4916	1	0.3218	1
BMI (kg/m^2)	0.9688	(0.862,1.0856)	0.5845	1	0.7214	1	0.4405	1
Eosinophils (0.01 G/l)	1.0085	(0.9725,1.0467)	0.6478	1	0.3394	1	0.2057	1
Histology: Adenocarcinoma	1.0345	(0.292,3.6658)	1	1	-	-	0.8389	1
Lymphocytes (G/l)	0.9549	(0.504,1.7948)	0.8838	1	0.7914	1	0.8044	1
Metformin: TRUE	0.3048	(0.0147,2.5168)	0.3570	1	-	-	0.4474	1
Monocytes (G/l)	0.6494	(0.1628,2.12)	0.4761	1	0.8090	1	0.7388	1
Neutrophils (G/l)	0.9501	(0.7949,1.1259)	0.5537	1	0.5047	1	0.1529	1
NSAR: TRUE	2.2609	(0.79116,6.9064)	0.1877	1	-	-	0.0884	1
PD-L1TC: 1.00-50.00%*	1.2587	(0.4163,3.8498)	0.6829	1	-	-	0.9115	1
PD-L1TC: >50%*	1.2121	(0.3578,4.1577)	0.7565	1	-	-	-	-
Sex: Woman	2.1477	(0.776,6.2671)	0.2003	1	-	-	0.1693	1
Smoker: TRUE	0.5813	(0.1115,2.5736)	0.7101	1	-	-	0.5897	1
Steroids: TRUE	0.8273	(0.3157,2.1478)	0.8092	1	-	-	0.8917	1
Treatment: ICI & Chemo	1.4899	(0.5772,3.9073)	0.4736	1	-	-	0.2024	1

OR: odds ratio, LR: likelihood ratio

Table S6: Results of all analyses with respect to development of immune-related adverse events (irAE) in the first-line patient group.

	Univariate Methods						Multivariate Methods	
	Univariate Logistic Regressions				Additional Tests		Classification Random Forest	
Variable	Estimate (OR)	95%-CI	Raw P-Value (LR/Fisher)	Adjusted P-Value (Holm)	Raw P-Value (Wilcoxon)	Adjusted P-Value (Holm)	Raw Impurity Importance P-Value	Adjusted P-Value (Holm)
Age (years)	1.0016	(0.9495,1.0578)	0.9526	1	0.9225	1	0.6102	1
Basophils (0.01 G/l)	0.9597	(0.7536,1.1982)	0.7209	1	0.7117	1	0.9844	1
BMI (kg/m^2)	0.8921	(0.7765,1.0111)	0.0747	1	0.1007	0.7050	0.0766	0.9958
Eosinophils (0.01 G/l)	0.9789	(0.9382,1.0178)	0.2898	1	0.2332	1	0.4224	1
Histology: Adenocarcinoma	1.6579	(0.4388,8.0794)	0.7390	1	-	-	0.3706	1
Lymphocytes (G/l)	0.6768	(0.3078,1.3368)	0.2719	1	0.5037	1	0.6598	1
Metformin: TRUE	not possible to fit				-	-	0.3430	1
Monocytes (G/l)	0.4011	(0.0652,1.5982)	0.2167	1	0.1849	1	0.0235	0.3290
Neutrophils (G/l)	1.0539	(0.8805,1.2607)	0.5578	1	0.4808	1	0.4420	1
NSAR: TRUE	1.4583	(0.4875,4.2529)	0.5799	1		-	0.6584	1
PD-L1TC: 1.00-50.00%*	0.8642	(0.2741,2.729)	0.8018	1	-	-	0.6085	1
PD-L1TC: >50%*	0.4148	(0.0945,1.5876)	0.2022	1	-	-	-	-
Sex: Woman	1.5692	(0.5433,4.4693)	0.4262	1	-	-	0.5164	1
Smoker: TRUE	4.0250	(0.6571,77.7154)	0.2515	1	-	-	0.4829	1
Treatment: ICI & Chemo	0.9500	(0.3504,2.6216)	1	1	-	-	0.6782	1

OR: odds ratio, LR: likelihood ratio

Table S7: Univariate logistic regressions on response within three months (Response3mt) and development of immune-related adverse events (irAE) with influential observations removed in the further-line patient group and the first-line patient group.

Patient Group	Response Variable	Predictor	Old Raw P-Value	Number of Observations removed	New Raw P-Value	New Adjusted P-Value (Holm)
Further Line	Response3mt	Basophils (0.01 G/l)	0.0027	3	0.00033	0.0056
		Eosinophils (0.01 G/l)	0.0215	1	0.00686	0.1098
	irAE	Basophils (0.01 G/l)	0.0049	4	0.00023	0.0038
First Line	irAE	BMI (kg/m^2)	0.0747	1	0.0341	0.4434
		Eosinophils (0.01 G/l)	0.2898	2	0.0369	0.4434
		Monocytes (G/l)	0.2167	2	0.0317	0.4434
		PDL1TC: >50%	0.2022	4	0.0173	0.2597

Table S8: Results of all analyses with respect to overall survival in the further-line patient group.

	Univariate Methods				Multivariate Methods	
	Univariate Cox Proportional Hazard Regressions				Survival Random Forest	
Variable	Estimate (HR)	95%-CI	Raw P-Value (LR)	Adjusted P-Value (Holm)	Raw Impurity Importance P-Value	Adjusted P-Value (Holm)
Age (years)	1.0086	(0.9868,1.0308)	0.4400	1	0.9750	1
Antibiotics: TRUE	1.2396	(0.8012,1.9176)	0.3350	1	0.6975	1
Basophils (0.01 G/l)	0.9157	(0.8276,1.0132)	0.0785	1	0.8453	1
BMI (kg/m^2)	0.9841	(0.9407,1.0295)	0.4813	1	0.9097	1
Eosinophils (0.01 G/l)	0.9960	(0.9848,1.0074)	0.4819	1	0.5649	1
Histology: Adenocarcinoma	0.7410	(0.4562,1.2035)	0.2361	1	0.2166	1
Lymphocytes (G/l)	0.7268	(0.524,1.008)	0.0475	0.7119	0.6327	1
Metformin: TRUE	0.7676	(0.3528,1.67)	0.4893	1	0.6126	1
Monocytes (G/l)	1.0227	(0.5245,1.9944)	0.9474	1	0.0453	0.7248
Neutrophils (G/l)	0.9284	(0.8433,1.0222)	0.1136	1	0.0716	1
NSAR: TRUE	0.7131	(0.4522,1.1247)	0.1404	1	0.4537	1
PD-L1TC: 1.00-50.00%*	0.7647	(0.4475,1.3067)	0.3221	1	0.2144	1
PD-L1TC: >50%*	0.3579	(0.1494,0.8575)	0.0101	0.1717	-	-
Sex: Woman	0.6541	(0.4032,1.0612)	0.0772	1	0.4078	1
Smoker: TRUE	0.4883	(0.2571,0.9274)	0.0441	0.7059	0.0490	0.7350
Steroids: TRUE	0.8592	(0.553,1.3349)	0.4988	1	0.8155	1
Treatment: ICI & Chemo	0.8512	(0.3698,1.9595)	0.6985	1	0.6633	1

HR: hazard ratio, LR: likelihood ratio

Table S9: Results of all analyses with respect to overall survival in the first-line patient group.

	Univariate Methods				Multivariate Methods	
	Univariate Cox Proportional Hazard Regressions				Survival Random Forest	
Variable	Estimate (HR)	95%-CI	Raw P-Value (LR)	Adjusted P-Value (Holm)	Raw Impurity Importance P-Value	Adjusted P-Value (Holm)
Age (years)	1.0271	(0.983, 1.0732)	0.2228	1	0.2776	1
Antibiotics: TRUE	1.6767	(0.7275, 3.8647)	0.2121	1	0.7729	1
Basophils (0.01 G/l)	1.0347	(0.8648, 1.2379)	0.7128	1	0.5817	1
BMI (kg/m^2)	1.0338	(0.9407, 1.1361)	0.4925	1	0.8990	1
Eosinophils (0.01 G/l)	0.9928	(0.9625, 1.024)	0.6444	1	0.7959	1
Histology: Adenocarcinoma	0.3273	(0.135, 0.7934)	0.0239	0.3579	0.0202	0.3030
Lymphocytes (G/l)	0.3912	(0.1984, 0.7714)	0.0031	0.0535	0.1721	1
Metformin: TRUE	1.3648	(0.3219, 5.7867)	0.6861	1	0.3794	1
Monocytes (G/l)	0.4635	(0.1304, 1.6469)	0.1892	1	0.7757	1
Neutrophils (G/l)	1.1255	(0.9943, 1.274)	0.0810	1	0.0088	0.1408
NSAR: TRUE	0.2478	(0.0738, 0.8325)	0.0078	0.1256	0.0336	0.4708
PD-L1TC: 1.00-50.00%*	0.9626	(0.4086, 2.268)	0.9306	1	0.7302	1
PD-L1TC: >50%*	0.4971	(0.1684, 1.4677)	0.1927	1	-	
Sex: Woman	0.8204	(0.344, 1.9564)	0.6505	1	0.9802	1
Smoker: TRUE	0.6963	(0.2393, 2.0258)	0.5240	1	0.6940	1
Steroids: TRUE	1.5603	(0.6771, 3.5957)	0.2843	1	0.9775	1
Treatment: ICI & Chemo	2.4608	(0.9909, 6.1112)	0.0420	0.5886	0.0989	1

*Note: The data for the analysis of PD-L1TC had 1 patient less than the other analyses

HR: hazard ratio, LR: likelihood ratio

Table S10: Univariate Cox proportional hazard regressions on overall survival with influential observations removed in the further-line patient group and the first-line patient group.

Patient Group	Predictor	Old Raw P-Value	Number of Observations removed	New Raw P-Value	New Adjusted P-Value (Holm)
Further Line	Histology: Adenocarcinoma	0.2361	3	0.0218	0.3489
	Neutrophils (G/l)	0.1136	1	0.0455	0.6364
	Smoker: TRUE	0.0441	1	0.0246	0.3693
First Line	Histology: Adenocarcinoma	0.0239	1	0.0053	0.0790
	Lymphocytes (G/l)	0.0031	1	0.0010	0.0166
	Neutrophils (G/l)	0.0810	2	0.0054	0.079
	NSAR: TRUE	0.0078	3	0.00002	0.0004

Table S11: Significant Cut-off values of continuous variables with respect to univariate logistic regressions on response within three months (Response3mt) and development of immune-related adverse events (irAE) in the further-line patient group and the first-line patient group.

Patient Group	Response Variable	Predictor	Estimated Cut-off (in one unit)	P-Value of Cut-off	P-Value Fisher (Binarized a.t. healthy range)
Further Line	Response3mt	Basophils (G/l)	0.02	0.0042	1
		Lymphocytes (G/l)	1.45	0.1118	0.0466
	irAE	Basophils (G/l)	0.05	0.0133	1
First Line	Response3mt	Neutrophils (G/l)	7.15	0.3855	0.0346
	irAE	Monocytes (G/l)	0.51	0.0266	0.3404

Table S12: Significant Cut-off values of continuous variables with respect to univariate Cox regressions on overall survival in the further-line patient group and the first-line patient group.

Patient Group	Predictor	Estimated Cut-off (in one unit)	P-Value of Cut-off	P-Value LR (Binarized a.t. healthy range)
Further Line	BMI (kg/m^2)	30.10	0.208	0.0748
	Lymphocytes (G/l)	1.45	0.1016	0.0415
First Line	Lymphocytes (G/l)	1.78	0.0286	0.0979
	Neutrophils (G/l)	7.15	0.0669	0.0415

LR: likelihood ratio
